# Same day comparison of PET/CT and PET/MR in patients with cardiac sarcoidosis

**DOI:** 10.1007/s12350-018-01578-8

**Published:** 2019-01-02

**Authors:** G. Wisenberg, J. D. Thiessen, W. Pavlovsky, J. Butler, B. Wilk, F. S. Prato

**Affiliations:** 1 Departments of Medicine, Medical Imaging, and Medical Biophysics, Western University, London, ON Canada; 2 Departments of Medical Biophysics, Medical Imaging and Physics and Astronomy, Western University, London, ON Canada; 3 Department of Medical Imaging, Western University, London, ON Canada; 4 Division of Nuclear Medicine, St. Joseph’s Hospital, London, ON Canada; 5 Lawson Health Research Institute, London, ON Canada; 6 MyHealth Centre, 21589 Richmond Street, Arva, ON N0M 1C0 Canada

**Keywords:** Cardiac sarcoidosis, positron emission tomography, magnetic resonance imaging, PET/CT, PET/MR, ^18^F-FDG

## Abstract

**Background:**

Inflammatory cardiac disorders, in particular, sarcoidosis, play an important role in left ventricular dysfunction, conduction abnormalities, and arrhythmias. In this study, we compared the imaging characteristics and diagnostic information obtained when patients were imaged sequentially with PET/CT and then with hybrid PET/MRI on the same day following a single ^18^F-FDG injection.

**Methods:**

Ten patients with known or suspected sarcoidosis underwent imaging in sequence of (a) ^99m^Tc-MIBI, (b) ^18^F-FDG with PET/CT, and (c) ^18^F-FDG with 3T PET/MRI. Images were compared quantitatively by determination of SUV_max_ and SUV on a voxel by voxel basis, and qualitatively by two experienced observers.

**Results:**

When both platforms were compared quantitatively, similar data for the evaluation of enhanced ^18^F-FDG uptake were obtained. Qualitatively, there were (1) several instances of normal perfusion with delayed enhancement and/or focal ^18^F-FDG uptake, (2) comparable enhanced ^18^F-FDG uptake on PET/CT vs. PET/MRI, and (3) diversity in disease patterns with delayed enhancement only, increased ^18^F-FDG uptake only, or both.

**Conclusion:**

In this limited patient study, PET/CT and PET/MR provided similar diagnostic data for ^18^F-FDG uptake, and the concurrent acquisition of MR images provided further insight into the disease process.

**Electronic supplementary material:**

The online version of this article (10.1007/s12350-018-01578-8) contains supplementary material, which is available to authorized users.

## Introduction

Imaging has assumed an important role in diagnosis and guiding of therapeutic decisions in the setting of known or suspected sarcoidosis and cardiac inflammatory disorders. Cardiac involvement with sarcoidosis can lead to left ventricular dysfunction, conduction disturbances, and ventricular arrhythmias,^1^–^5^ related to active inflammation and/or scar.^6^ The presence of late Gadolinium enhancement in sarcoid patients confers a much higher risk of malignant ventricular arrhythmias.^7^ Cardiac involvement in sarcoidosis may affect up to 25% of patients based on pathology data.^8^,^9^

Co-incident with the understanding of the importance of sarcoidosis has been the development of hybrid imaging systems which combine Positron Emission Tomography (PET) with either Computed tomography (CT) or Magnetic Resonance Imaging (MRI). PET is ideally suited to assess active macrophage-mediated inflammation using ^18^F-FDG.^10^,^11^ Both M1 (pro-inflammatory macrophages) and M2 (anti-inflammatory macrophages) actively sequester ^18^F-FDG, 12,13 with evidence of a 20:1 ratio favoring sequestration by M1 macrophages.^14^ Inflammation can be detected by ^18^18F-FDG imaging, provided myocardial ^18^F-FDG uptake is selectively suppressed. When ^18^F-FDG-PET is combined with CT, the CT provides attenuation correction, an anatomic reference, and unsuspected noncardiac findings. MRI not only provides the above, but also tissue characterization for the detection of scar and edema. When PET and MRI are combined, their geographic relationship (scar/edema and inflammation) can be ascertained.^15^ As such, hybrid PET/MRI may be more useful in managing individual patients, and on a broader scale, understanding the relationship and evolution of these processes may enhance our knowledge of the pathophysiology of sarcoidosis.

As a preliminary attempt to compare image characteristics and diagnostic details of PET/CT and PET/MRI, we performed a pilot study of 10 patients with known or suspected cardiac sarcoidosis.^10^ All patients were imaged initially with PET/CT and then PET/MRI on the same day, following a single bolus injection of ^18^F-FDG.

## Methods

### Patient Selection

Patients participated if they were to undergo an ^18^F-FDG PET/CT scan for assessment of active cardiac inflammation when there was a suspicion of sarcoidosis or in patients with established biopsy proven extracardiac sarcoid. Patients were consecutive patients who agreed to participate in the protocol and who did not have a cardiac defibrillator. Of 53 scans ordered during that time interval, 37 patients had defibrillators, and three declined participation, and three could not be imaged for technical reasons (size, body piercing). The remaining ten consented and form the cohort of the study.

### Imaging Protocols

**Figure 1:**
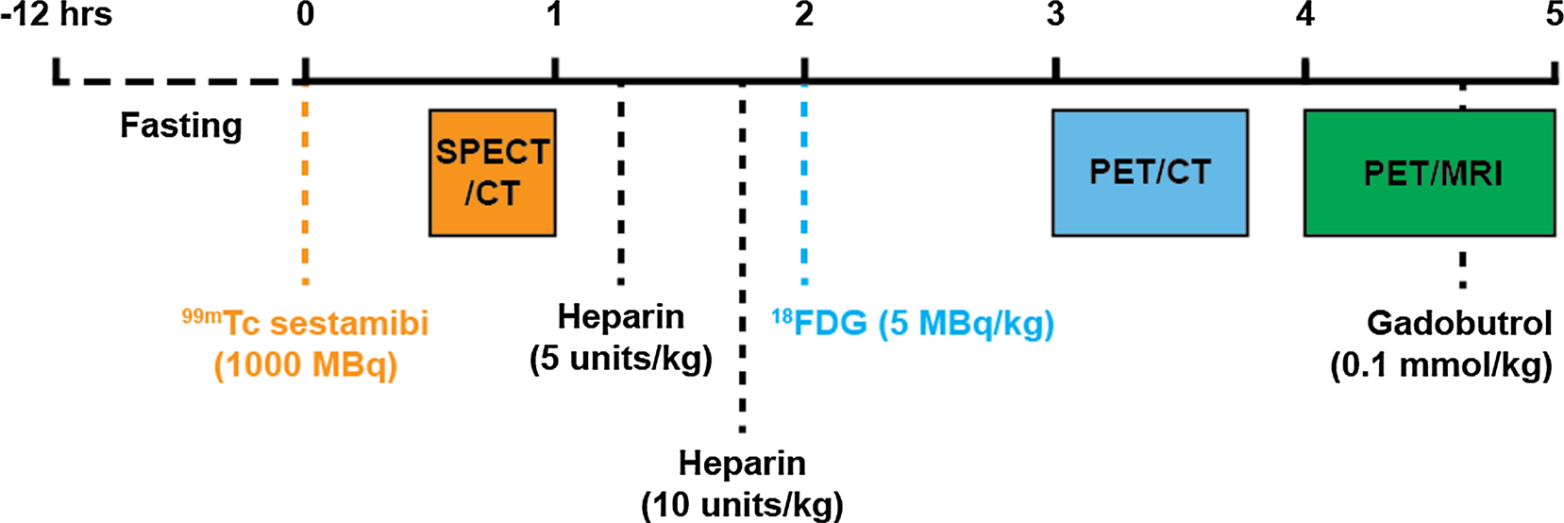
Schematic presentation of the imaging protocol

A summary of the imaging protocol is presented as Figure 1. Patients were asked (a) to fast for 12 hours prior to scanning, (b) to follow a high fat, low-carbohydrate, protein-permitted diet the day before the scan, and (c) to present for the scanning session well hydrated.

For the perfusion study, SPECT imaging (Siemens Symbia T6 SPECT/CT) was performed using technetium (^99m^Tc) sestamibi. Approximately 1000 MBq was injected intravenously. Scanning began after a delay of 30-60 minutes for blood clearance of the tracer. Acquisitions consisted of 60 static projections, 25 seconds/projection, reconstructed with an ordered subset expectation maximization (OSEM) algorithm, Butterworth filter (0.4 cycles/cm cutoff), and CT-based attenuation correction. Tomographic images were presented as polar maps using the Emory Cardiac Toolbox on a GE Xeleris workstation for review. Perfusion was only assessed in this manner. There was no PET perfusion study performed in these patients.

One hour prior to the scheduled scan, ^18^F-FDG [5 MBq/kg injected intravenously (< 550 MBq(15 mCi) maximum] was administered. Unfractionated Heparin (5 units/kg) was given intravenously 45 minutes before ^18^F-FDG, followed by an additional dose of Heparin (10 units/kg) 30 minutes after the first injection (or 15 minutes prior to ^18^F-FDG administration).

^18^F-^18^F-FDG whole-body (1-3 minutes/bed position) and thorax-only (10 minutes each) PET images (PET_WBCT_ and PET_ThoCT_) were acquired on a PET/CT (GE Discovery VCT). PET/CT included a low-dose CT for attenuation correction (140 kVp, variable mA). PET from the PET/CT was reconstructed using the vendor-provided OSEM algorithm with an axial resolution of 5.47 mm and slice thickness of 3.27 mm.

Shortly after PET/CT, patients had thorax-only PET/MRI (PET_MR_) acquired on a Siemens Biograph mMR in a single bed position with a 3 Tesla magnet. A 25 minute PET scan was acquired during the first half of the PET/MR imaging session. PET images were reconstructed using the vendor-provided OSEM algorithm with 3 iterations, 21 subsets, an axial resolution of 2.09 mm, slice thickness of 2.03 mm, and 4 mm Gaussian filter.

MRI acquisitions during the PET/MRI scan included the vendor-provided 2-point Dixon MR-based attenuation correction (MRAC) at the start of each PET acquisition, thoracic T2-weighted coronal and axial half-fourier acquisition single-shot turbo spin echo (HASTE), breath-hold cine images using fast imaging with steady state precession (TrueFISP), and turbo inversion recovery magnitude (TIRM) images. Following a bolus injection of 0.1 mmol/kg gadobutrol (Bayer Gadovist^®^), late gadolinium enhancement (LGE) phase-sensitive inversion recovery (PSIR) and 3D inversion-recovery gradient-echo (3D-LGE) images were acquired to image fibrosis in the left ventricle.

## Image Analysis

### Clinical

All images were reviewed in a nonblinded fashion by a dual-certified nuclear medicine/radiologist (WP) and cardiologist (GW).

Initially, all the individual perfusion and corresponding ^18^F-FDG PET/CT scans were reviewed. The presence or absence of perfusion defects, and the extent, and location of such defects were determined by visual analysis. Then, the presence of enhanced ^18^F-FDG uptake was also established (only if it was regional, and significantly greater than blood pool activity). Diffuse uptake was suggestive of inadequate myocardial suppression.

Then, the PET/MRI images were reviewed on a separate day. The presence and extent of delayed enhancement was determined initially on the MR images. Following this, enhanced ^18^F-FDG uptake on the PET/MRI images was determined through visual analysis. The PET and MRI images were then fused, and the relationship of any enhanced ^18^F-FDG signal to the delayed enhancement was determined. LV wall motion was evaluated qualitatively for selected patients by a single observer (GW).

### Quantitative

Standardized uptake values (SUV) were calculated using the injected dose, body weight, and decay-corrected time of injection. After determining the maximum SUV (SUV_max_) in the thoracic region (avoiding uptake in the liver and spine), regions of interest with elevated uptake were defined using a threshold equal to 0.5 × SUV_max_ (3D Slicer 4.6.2). This was meant to determine the metabolically active volume (MAV) while accounting for variations in uptake over time and between patients. In addition, MAVs were calculated using fixed thresholds at SUV > 2.5 (MAV 2.5) and SUV > 4 (MAV 4).

Left-ventricular end diastolic volumes (EDV), end systolic volumes (ESV), stroke volume (SV), and ejection fractions (LVEF) were determined using threshold-based segmentation with manual adjustments (as required) in short-axis cine images spanning the left ventricle (3D Slicer 4.6.2).

All measurements were tested for normality using the Shapiro-Wilk test, where the null hypothesis is a normal distribution and *P* ≤ 0.05 indicates data is not normally distributed.

In order to demonstrate the quantitative accuracy of PET_MR_ vs PET_CT_, linear regression and Pearson correlation coefficients were calculated for SUV_max_ values from both PET_MR_ vs PET_WBCT_ and PET_MR_ vs PET_ThoCT_. A two-tailed paired *t* test was used to compare SUV_max_ values from both the PET/CT and PET/MRI. To measure voxel-wise correlation between SUV values, PET_ThoCT_ was manually aligned to PET_MR_ by a dual-certified PET/MR technologist with additional guidance provided by both anatomical CT and MR images (3D Slicer 4.6.2). PET_MR_ was then resampled to the PET_ThoCT_ resolution and both PET volumes were cropped to a volume surrounding the heart before scatter and Bland-Altman plots were generated using the aligned PET_ThoCT_ and PET_MR_ voxel values. Linear regression and Pearson correlation correlations were also calculated between the LVEF and the SUV_max_ and the three estimates of MAV.

### Review of Prior MRI and/or CT Imaging

If available, the results of prior studies were reviewed (in many cases there was a 2 month or greater time interval). All of these scans were performed on 1.5 Tesla systems.

## Results

A summary of patient characteristics are provided in Table 1, and of the MRI acquisition parameters in Table 2.

**Table 1:** Summary of patient characteristics

Patient	Sex	Age	Weight (kg)	Biopsy	Prior history
1	M	38	90.7	NA	Nonischemic cardiomyopathy
2	F	66	56.7	NA	Nonischemic cardiomyopathy
3	F	65	69.9	+	Pulmonary sarcoid
4	M	69	122.0	NA	Nonischemic cardiomyopathy
5	M	48	77.1	+	Mediastinal sarcoid with pericarditis
6	F	59	107.5	NA	Pulmonary sarcoid
7	M	70	89.8	+ (remote)	Pulmonary sarcoid
8	F	68	78.0	NA	Syncope, nonsustained VT
9	M	63	108.9	NS	Pulmonary and hilar adenopathy
10	M	58	95.7	+ (renal)	Pulmonary fibrosis

*NA*, not acquired; *NS*, nonspecific; +, positive biopsy

**Table 2:** MRI acquisition parameters

	FOV (mm^3^)	Spatial resolution (mm^3^)	Motion correction	Acquisition time/view	Views
MRAC	500 × 328 × 400	2.6 × 2.6 × 3.2	None	18s	3D
HASTE	440 × 440 × 225	1.72 × 1.72 × 6	BH	5s	Coronal, Axial
TrueFISP	286 × 340 × 6	1.33 × 1.33 × 6	BH, ECG	10s	2C, 4C, SA
TIRM	265 × 340 × 8	1.33 × 1.33 × 8	BH, ECG	10s	2C, 4C, SA
PSIR	217 × 290 × 8	1.13 × 1.13 × 8	BH, ECG	6s	2C, 4C, SA
3D-LGE	250 × 320 × 130	0.625 × 0.625 × 0.9	NE, ECG	6–8 min	3D

*MRAC*, MR-based attenuation correction; *HASTE*, half-fourier acquisition single-shot turbo spin echo; *TrueFISP*, true fast imaging with steady-state precession; *TIRM*, turbo inversion recovery magnitude; *PSIR*, phase-sensitive inversion recovery; *LGE*, late gadolinium enhancement; *FOV*, field of view; *BH*, breath-hold acquisition; *NE*, navigator echo for respiratory gating; *ECG*, electrocardiogram for cardiac gating or triggering; *2C*, 2-chamber view; *4C*, 4-chamber view; *SA*, short-axis stack spanning the left ventricle from apex to base

### Clinical Comparison

**Figure 2:**
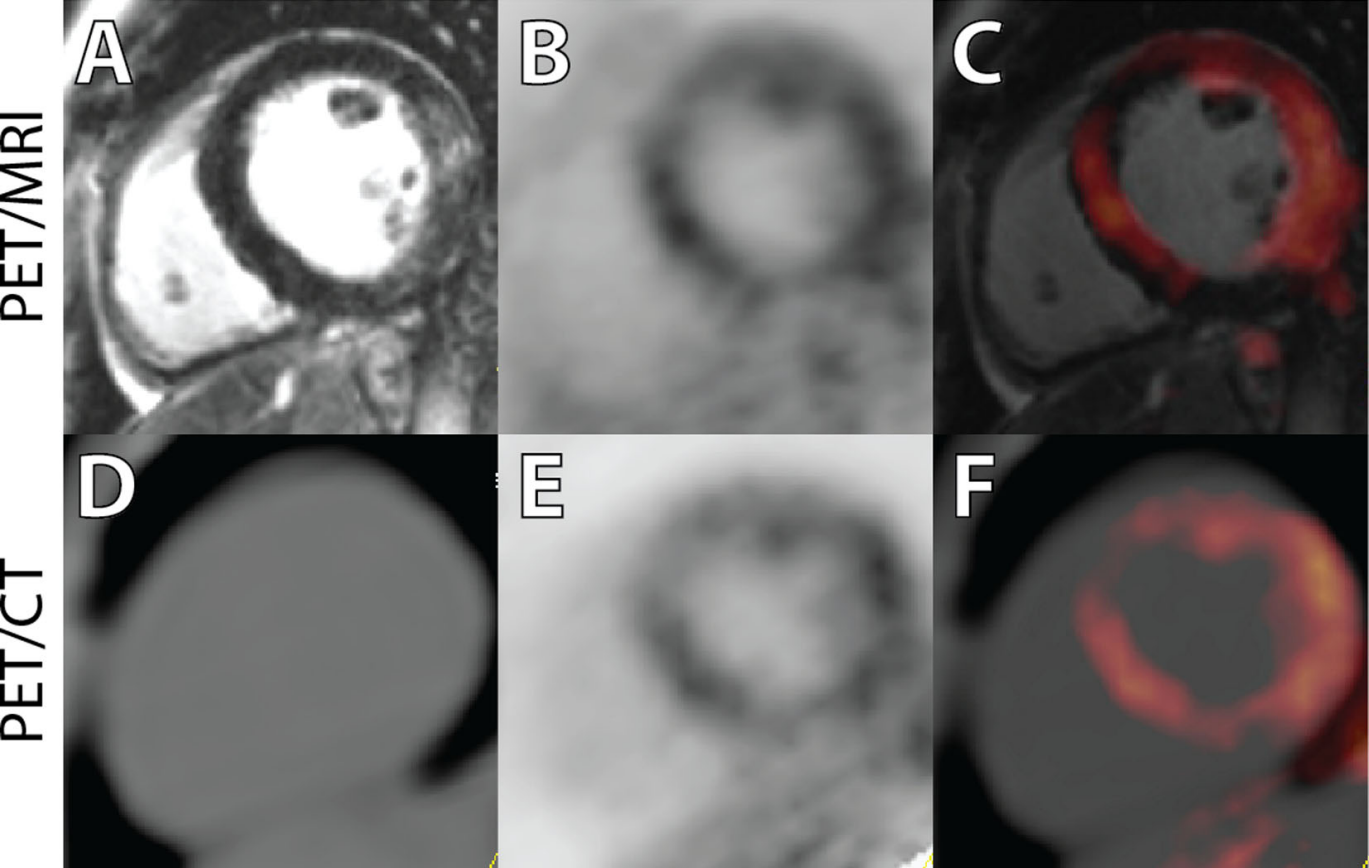
Images presented from Patient 1 with PET/MR (**A**–**C**) and PET/CT (**D**–**F**). In this patient, note the enhanced signal seen on the delayed enhancement MR images in the lateral wall (**A**), and the enhanced ^18^F-FDG uptake in the septal, anterior, and lateral regions on both the PET/CT and PET/MR images (**C**, **F**). Although this extensive uptake of ^18^F-FDG could be interpreted as poor suppression, an increase in ^18^F-FDG in the hilum and great vessels (not shown in these images) is supportive of this interpretation. This patient had an ejection fraction of 49% with mild global hypokinesis. There were no regional wall motion abnormalities

**Figure 3:**
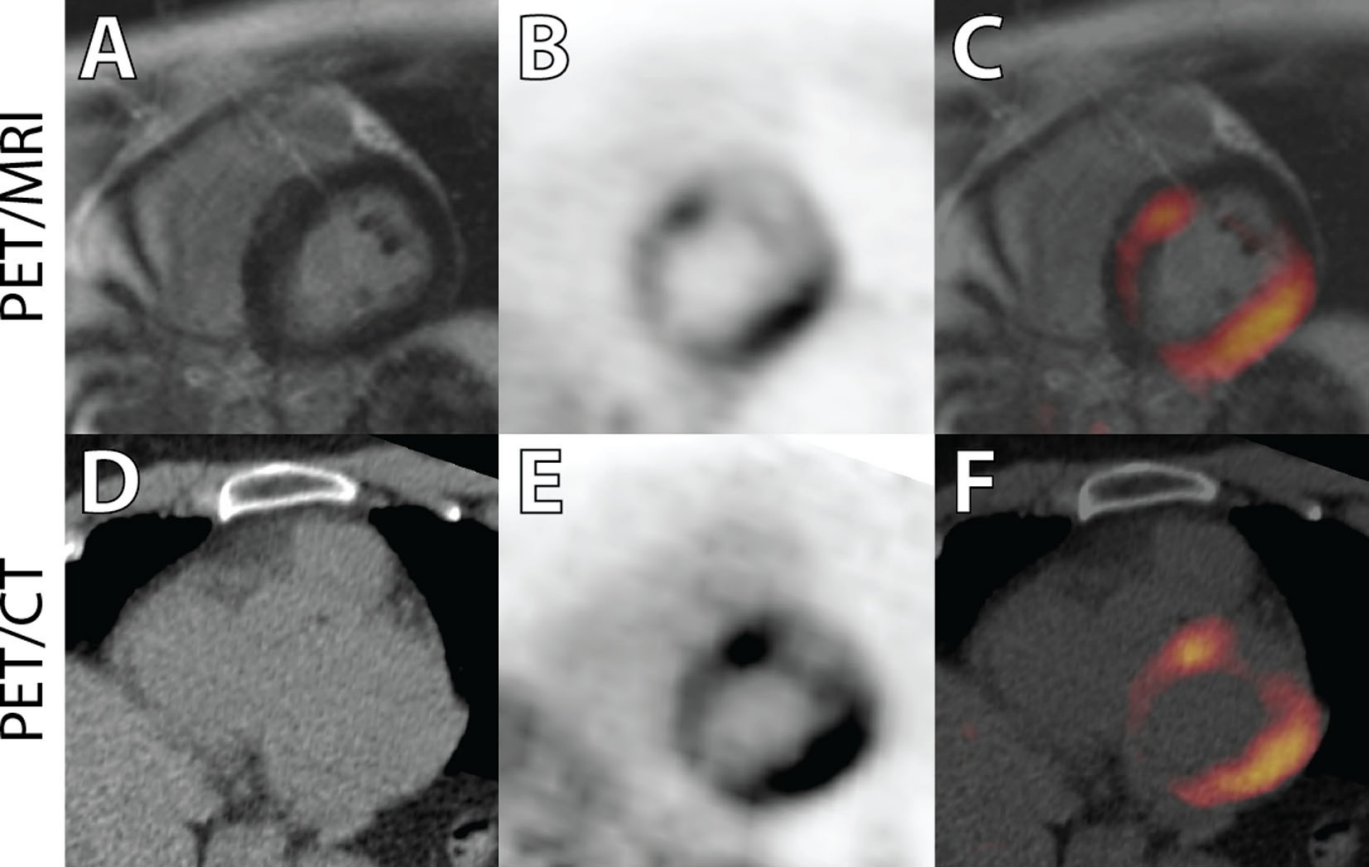
Images presented from patient 7 in a similar format as for Fig. 2. In this image, there is no area of signal enhancement on the delayed enhancement MR image (**A**). However, there is a marked increase in uptake, essentially transmural in the lateral wall and anterior septum (**B**, **C**). There is somewhat greater definition as to the extent of this uptake seen on the PET/MR images vs the PET/CT images (**E**, **F**). This patient’s ejection fraction was 61% with moderate septal hypokinesis

**Figure 4:**
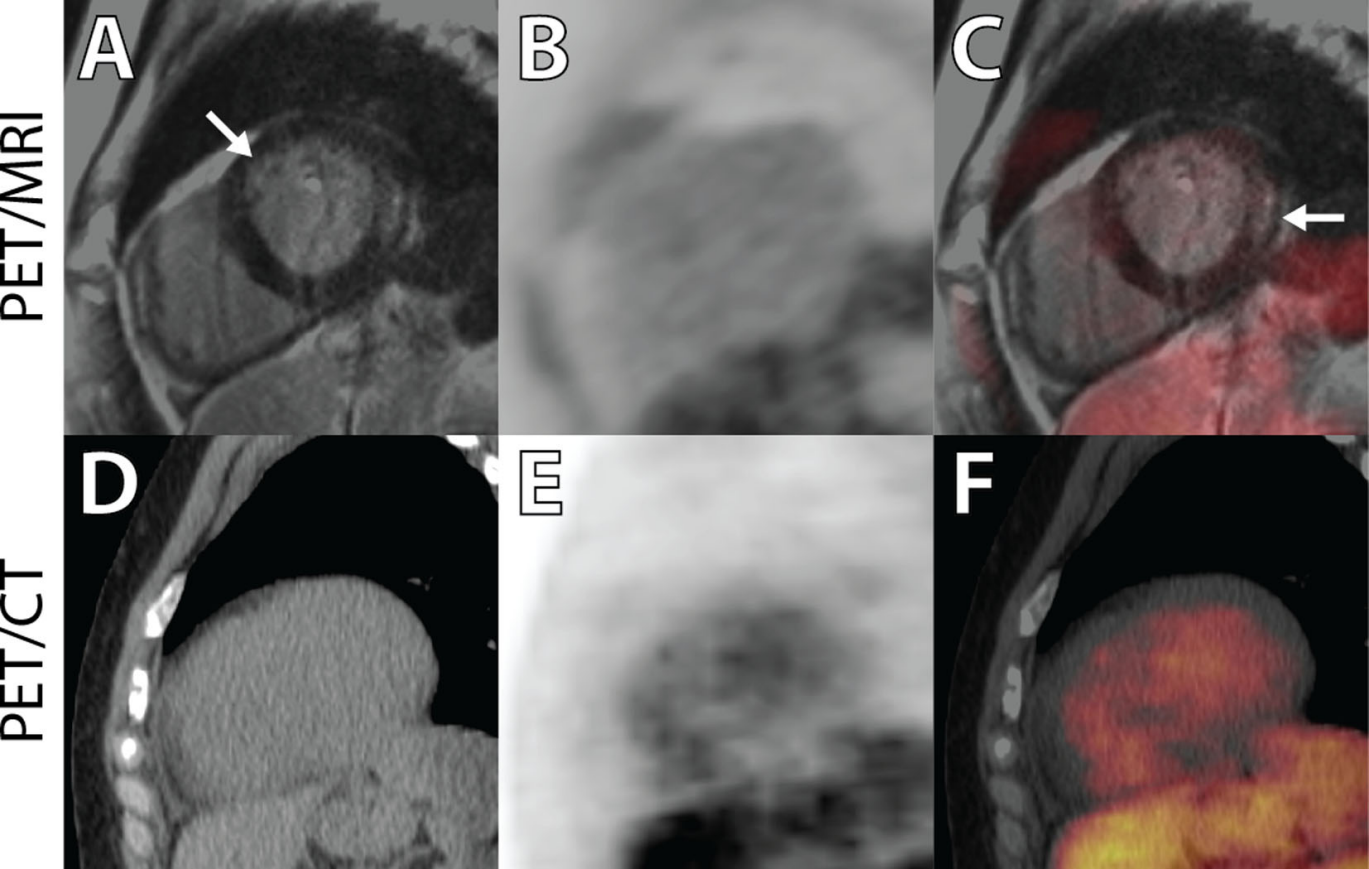
Images from patient three. In this case, an enhanced signal is seen on the delayed enhancement MR images in both mid-septum and lateral regions (**A**) [arrows]. There is no corresponding increase in ^18^F-FDG signal on the PET images (**B**, **C**). Note the increased blood-pool activity on the PET/CT images that were taken approximately 2 h earlier (see Table 4), from the same single injection of ^18^F-FDG (**E**, **F**). The ejection fraction for this patient was 52% with mild inferoseptal hypokinesis

The results of the clinical comparison are provided in Table 3 and Figures 2, 3, and 4. From these comparisons, we obtain the following observations:

**Table 3:** Summary of SPECT, PET/CT, and PET/MRI readings

Patient	SPECT	PET_CT_	PET_MR_	MRI
1 (Fig. 2)	NP	Increase in basal septum and lateral wall. Extracardiac uptake in hilum and great vessels	Same as PET_CT_	Diffuse patchy scar in anterior, lateral, and inferior walls
2	NP	Slight increase in lateral wall. Increased thyroid uptake	More obvious and extensive uptake in lateral wall, ascending aorta, esophagus, and right shoulder	Extensive subendocardial scar in inferior septum and mid-inferior wall
3 (Fig. 4)	Anteroapical PD	No cardiac uptake. Uptake in hilar nodes and thyroid	No cardiac uptake. Increased ^18^F-FDG in nodes, esophagus, aorta, and left subclavian	*Extensive mid-inferior and inferior septal scar. Transmural in some regions*
4	Anterior and lateral PD	Increased ^18^F-FDG matching PD and in inferior wall	More extensive uptake in myocardium	*Mid-wall linear scar in septum with no* ^*18*^*F-FDG uptake in that region*. Extensive edema involving anterior wall, septum, and inferior wall
5	DP in anterior wall and septum	Increased ^18^F-FDG in basal lateral wall. Increased uptake in mediastinal nodes	Additional ^18^F-FDG uptake in septum, apical and lateral wall. Similar nodal uptake	*No Gd-enhancement*
6	NP	Increased ^18^F-FDG in basal anterior wall and inferior wall. Multiple extracardiac sites including hilar, mediastinal nodes, and spine	Similar but more extensive uptake in septum and lateral wall. Similar nodal uptake	*Distal anterolateral wall scar, distal septal scar with* ^*18*^*F-FDG surrounding both scar regions*
7 (Fig. 3)	NP	Enhanced ^18^F-FDG uptake in basal septum and lateral wall	More extensive uptake in lateral wall. Increased uptake in lung	Patchy distal septal enhancement
8	NP	Normal ^18^F-FDG	Normal ^18^F-FDG	Lateral wall subendocardial scar
9	NP	No cardiac uptake. Extracardiac uptake in hilum, lung, and aorta	Same as PET/CT	Small inferolateral scar
10	NP	No cardiac uptake. Extracardiac uptake in lungs, hilar, and mediastinal nodes	Same as PET/CT	Localized lateral subendocardial scar

*Italics* indicates MRI findings that added significantly to the PET/MR reading

*NP*, normal perfusion, *PD*, perfusion defect, *DP*, decreased perfusion; *Gd*, Gadobutrol

1. On comparison of ^18^F-FDG uptake on the PET/CT vs. PET/MRI, the presence/absence and the location of enhanced uptake were similar in all patients. The degree of contrast between enhanced uptake and background was greater on PET/MRI in all subjects with a positive ^18^F-FDG scan.
2. Although perfusion abnormalities were seen in some patients, there were several instances of completely normal perfusion scans with enhanced regional and/or patchy ^18^F-FDG uptake (pts 1, 2, 6, and 7).
3. Of the ten patients, eight had previously undergone a dedicated cardiac MRI on a 1.5 Tesla system. In all cases but one, there was a pattern of enhanced uptake suggestive of regional scar, with concordance between the MR findings on the dedicated MR and the PET/MR study.
4. There were considerable divergence in the extent and patterns of sarcoid involvement, ranging from the presence of myocardial scar only, in pts 3, 8, 9,10 (Figure 4), inflammation only (see Figure 3), or the two patterns present concurrently (Figure 2). In addition, several patients had significant extracardiac inflammation (hilar and mediastinal lymph nodes/great vessels) as indicated in Tables 1 and 3.

### Quantitative Analysis

**Figure 5:**
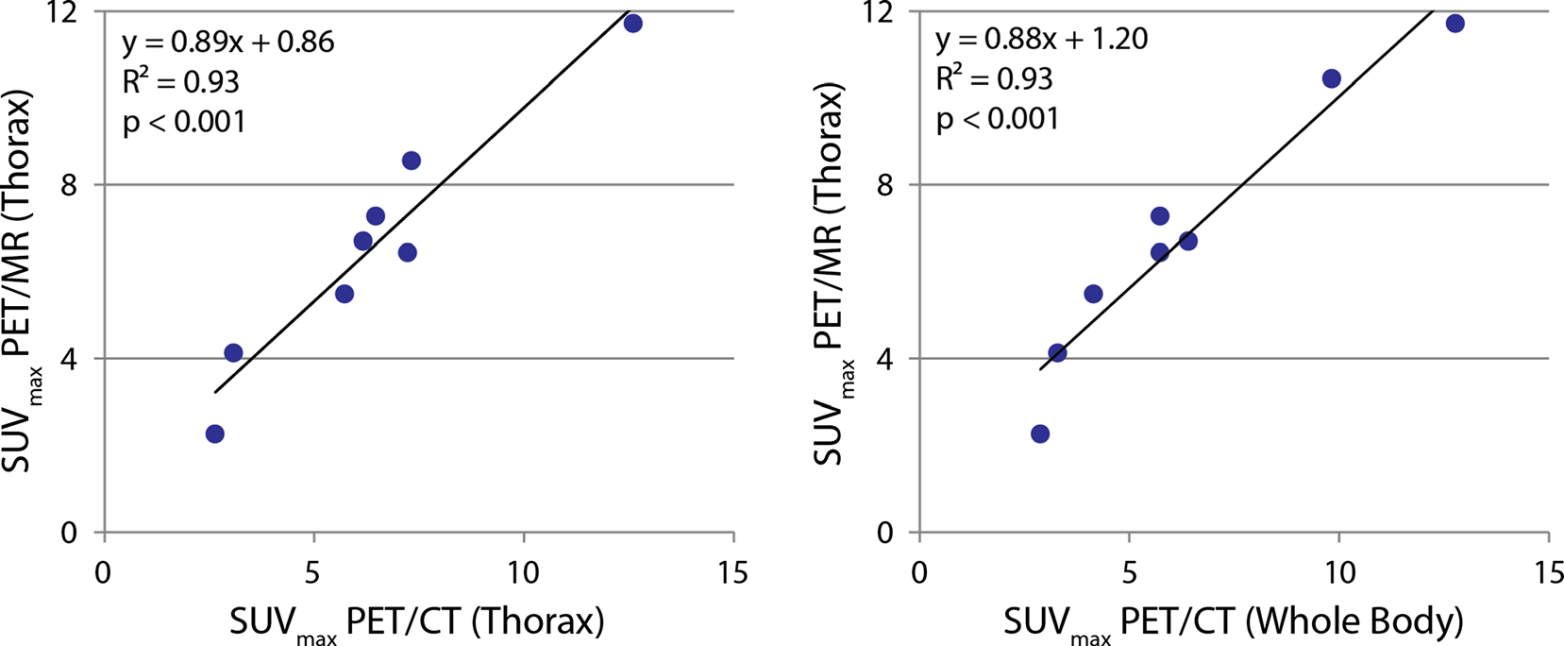
SUV_max_ values determined with PET/MR strongly correlating with both SUV_max_ values determined with PET/CT acquired in a single bed position (left) and whole body (right). *Note* Patient eight was removed from comparison due to normal uptake in both PET/CT and PET/MR. One SUV_max_ value was outside the axial FOV in the PET/CT (thorax) acquisition, and was removed from the comparison on the left. PET/CT (whole body) was not completed in another patient, and was removed from the comparison on the right

**Figure 6:**
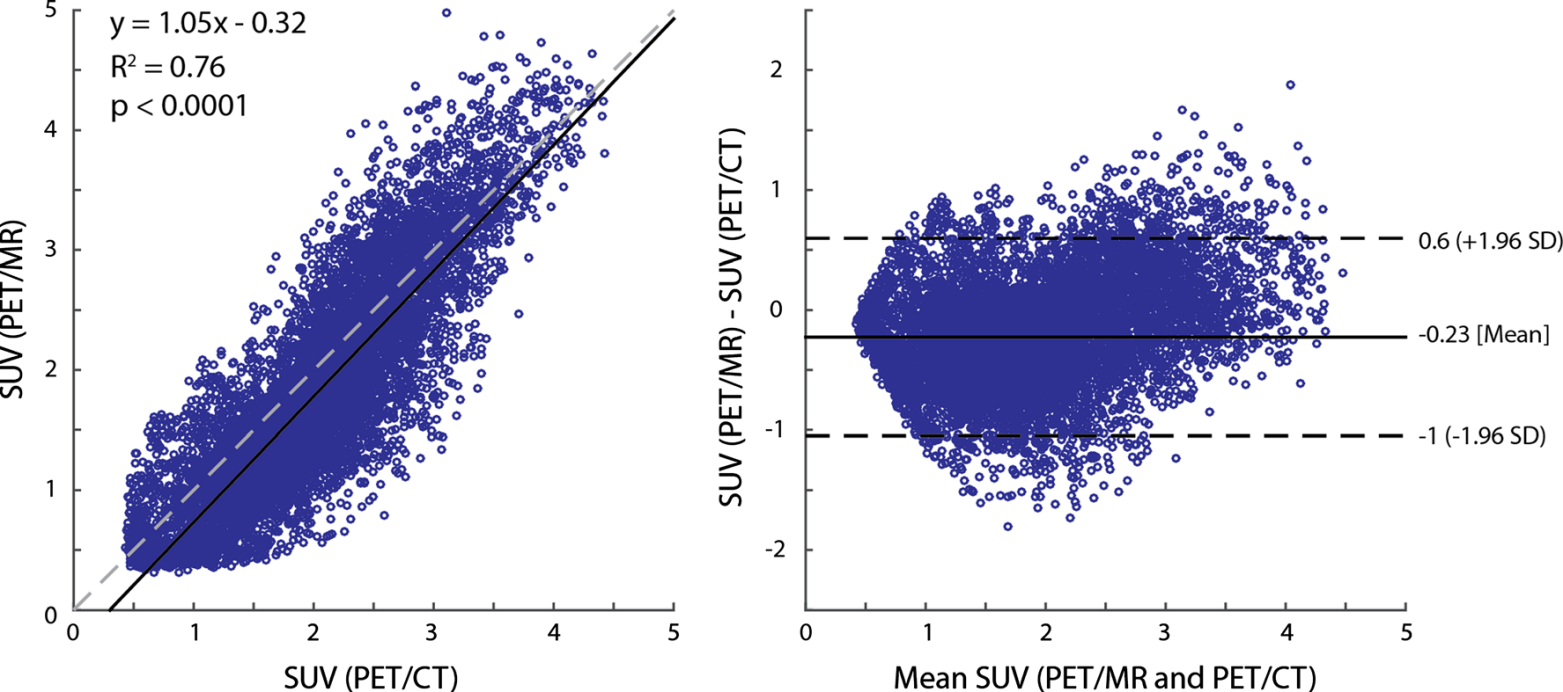
Voxel-wise comparison of co-registered and resampled SUV values from one PET/MR and PET/CT acquisition. Although correlation is strong, it is important to note that different times of acquisition and small registration errors will influence the correlation coefficient. Segmentation of the myocardium in the left ventricle was performed using the 3D-LGE MR data in this patient; however, this was not replicable due to patient motion in subsequent experiments

SUV_max_ values in the thoracic region determined by both PET/CT and PET/MRI were strongly correlated (Figure 5). Voxel-wise correlation of SUV values cropped to a region surrounding the heart in a single patient was also strong, but was influenced by the accuracy of the image registration and changing tissue uptake and blood concentration over time (Figure 6).

^18^F-FDG-PET results from all three acquisitions (PET_WBCT_, PET_ThoCT_, and PET_MR_) are summarized in Table 4. SUV_max_ values were normally distributed with no significant differences between the PET data collected with PET/CT and PET/MR (Figures 5, 6).

**Table 4:** Summary of PET results

Pt	Dose (MBq)	Start time after injection (m)			SUV_max_		
		PET_WB_	PET_Tho_	PET_MR_	PET_WB_	PET_Tho_	PET_MR_
1	300	136	146	210	6.4	6.2	6.7
2	260	62	94	133	2.9	2.6	2.3
3	370	54	85	128	5.7	7.2	6.4
4	628	56	94	139	3.3	3.1	4.1
5	407	NA	94	131	NA	7.3	8.5
6	528	59	91	133	5.7	6.5	7.3
7	371	81	115	162	4.1	5.7	5.5
8	415	79	93	141	N	N	N
9	542	111	125	165	12.8	12.6	11.7
10	476	76	83	112	9.8	6.5	10.4
Mean	430	79	102*	145	6.3	6.4	7.0
Std Dev	114	28	20	28	3.4	2.9	2.9

Pt	Dose (MBq)	MAV (cc) SUV > 0.5 x SUV_max_			MAV (cc) SUV > 2.5			MAV (cc) SUV > 4		
		PET_WB_	PET_Tho_	PET_MR_	PET_WB_	PET_Tho_	PET_MR_	PET_WB_	PET_Tho_	PET_MR_
1	300	473	247	182	1000	504	361	129	86	90
2	260	–	–	–	1.3	0.4	–	–	–	–
3	370	59	12	37	93	34	78	13	7.6	16
4	628	1325	236	567	19	10	182	–	–	0.1
5	407	NA	26	23	NA	118	140	NA	19	28
6	528	96	28	33	212	88	136	15	7.5	20
7	371	200	27	37	75	51	53	0.8	3.3	5.4
8	415	N	N	N	N	N	N	N	N	N
9	542	32	28	31	237	133	152	91	64	67
10	476	15	13	13	108	40	87	25	3.2	23
Mean	430	314*	77*	190*	218*	109*	149	46	27*	31
Std Dev	114	473	102	67	327	155	96	52	34	31

*NA*, not acquired; *N*, normal uptake; *PET*_*WB*_, whole-body PET from PET/CT; *PET*_*Tho*_, thorax-only PET from PET/CT; *PET*_*MR*_, thorax-only PET from PET/MR

*P ≤ 0.05 in Shapiro-Wilk normality test, where null-hypothesis (*P* > 0.05) is that the data is normally distributed

**Figure 7:**
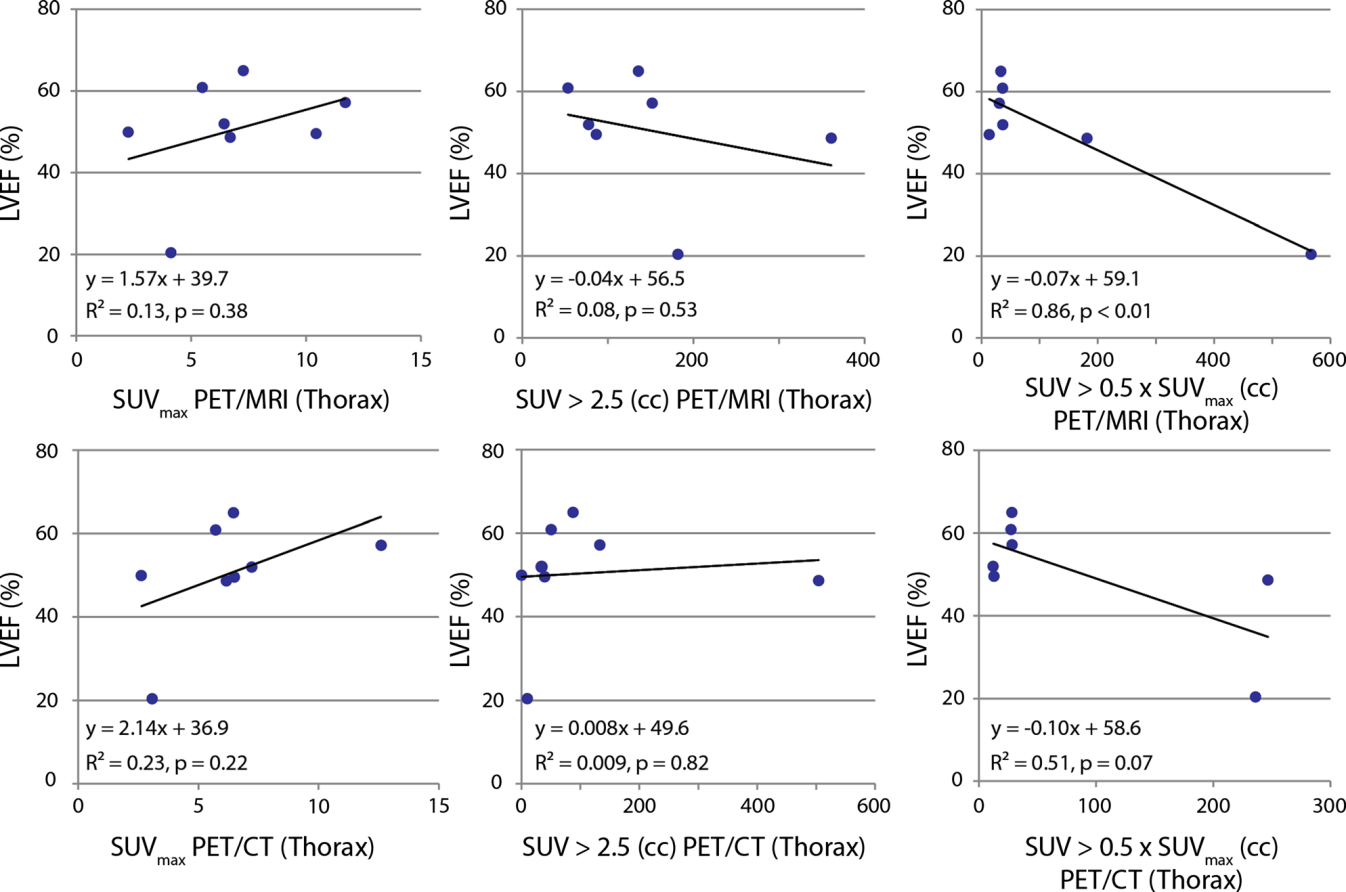
Left: weak correlation between LVEF and SUV_max_. One advantage of PET/MR is the ability to measure functional MRI simultaneously with metabolic PET. Although these results should be interpreted with caution, we see an increased correlation, particularly in the PET/MR, between LVEF and metabolically active volumes in the thoracic region defined using a threshold of SUV > 2.5 (middle) and SUV > 0.5 × SUV_max_ (right)

Measurements of cardiac function are summarized in Table 5. Simultaneous acquisition of PET and MRI allowed for correlation of LVEF with PET measurements. Correlation between LVEF and SUV_max_ was weak; however, there does appear to be increased correlation in the PET/MR between LVEF and MAV (Figure 7).

**Table 5:** Summary of functional MRI results

Patient	ESV (cc)	EDV (cc)	SV (cc)	LVEF (%)
1	72.8	141.7	68.9	48.6
2	28.0	55.9	27.9	49.9
3	68.5	142.6	74.1	51.9
4	223.1	280.3	57.1	20.4
5	NA	NA	NA	NA
6	31.4	89.5	58.1	64.9
7	31.3	79.9	48.6	60.8
8	61.5	119.7	58.2	48.6
9	45.0	105.2	60.1	57.2
10	58.6	116.2	57.5	49.5
Mean	68.9*	125.7*	56.7	50.2*
Std Dev	60.2	64.5	13.1	12.6

*EDV*, end diastolic volume; *ESV*, end systolic volume; *SV*, stroke volume; *LVEF*, left ventricular ejection fraction; *NA*, not acquired

*P ≤ 0.05 in Shapiro-Wilk normality test, where null-hypothesis (*P* > 0.05) is that the data is normally distributed

## Discussion

Imaging plays an important role in the diagnosis of cardiac inflammatory disorders, especially sarcoidosis. However, there are no universal guidelines as to which imaging tests should be performed routinely for initial screening in the absence of biopsy evidence. The most recent guidelines from 2014^16^ have suggested that it is *probable* that there is cardiac sarcoid if (a) there is biopsy evidence of the disease in an extracardiac site, **and** (b) one or more clinical criteria are met which include characteristic patterns for sarcoid on either MRI, ^18^F-FDG-PET, or gallium imaging, and other causes for these manifestations have reasonably been excluded. As such, the clinician is uncertain as to subjecting the patient to one or both imaging modalities, as MRI and PET focus on different aspects of the disease (MRI preferentially on scar/edema and ^18^F-FDG PET on macrophage-related inflammation). Combining both modalities simplifies decision making, but with the added expense of the hybrid unit. However, if both ^18^F-FDG-PET and 3T LGE-MRI are ultimately needed, the total cost of performing these separately exceeds the cost of a single hybrid PET/MRI examination.

Our study has provided evidence that, strictly from an imaging perspective, as expected, nothing is lost when combining the two imaging modalities. In all cases, PET/MRI provided diagnostic quality ^18^F-FDG images that were at least equal to or better than those obtained with either the PET/CT scan, and subjectively crisper MRI images than those obtained on a 1.5 T dedicated unit. If anything, the ^18^F-FDG images on the PET/MRI unit demonstrated, subjectively, improved image quality, with better definition of enhanced ^18^F-FDG uptake. However, this may have been related to the fact that all the PET/MRI studies were performed after the PET/CT study, with greater clearance of tracer from blood, and that a longer acquisition time was used (25 vs 10 minutes) to compensate for decay.

Beyond its use for diagnostic purposes, imaging can provide prognostic information,which may be useful in guiding therapy, eg. immunosuppressive treatment, and/or an implantable defibrillator. An abnormal ^18^F-FDG-PET scan is associated with a greater risk of ventricular arrhythmias and death,^5^ as is delayed enhancement.^7^ Also, using serial examinations following treatment, a reduction in inflammation is associated with improved left ventricular function and prognosis,^17^ although we did not see any correlation between SUV_max_ and LVEF in our study. Two recent studies have demonstrated divergent results with regards to the association of image findings with prognosis. A study reported by Vita showed that enhanced ^18^F-FDG uptake did not add prognostic value when combined with late gadolinium enhancement.^18^ However, a study by Wicks did suggest added value when the two modalities are combined.^19^

Although there was increased correlation between LVEF and metabolically active volumes in the thoracic region, given the small sample size and possible binomial distribution in some of the scatter plots, these results should be interpreted with caution. Nevertheless, this demonstrates the added value of acquiring PET and MRI data within the same system and at the same time.

## New Knowledge Gained

PET/MRI provides high-quality images in the evaluation of patients with suspected/known inflammatory disorders and potentially may be the diagnostic test of choice for evaluating these patients.

## Limitations

T2/edema measurements were attempted by MRI but, as the patients were too exhausted, image quality was insufficient for analysis. Similarly, quantitative analysis of some MRI datasets was not possible due to motion artifacts. Further, the small sample size limits the ability to generalize the results, although the principal focus of the study was to compare the imaging characteristics of the two devices (PET/CT vs. PET/MR). The scans were interpreted in an unblinded fashion, but the quantitative comparison would not have been affected by this issue. Further, perfusion was assessed using Tc-based SPECT and not with a PET perfusion tracer. Finally, PET/MR was always delayed compared to PET/CT, changing the time from injection to imaging and decreasing the amount of cavitary blood activity and potentially affecting the SUV values.

Many potential patients were excluded from participation in this study because of the presence of implantable defibrillators. This will be an ongoing limitation of this hybrid technology unless the patient had an MRI-compatible unit implanted.

## Conclusions

This study provides a small sample of the imaging capabilities and the potential role in patient management of advanced hybrid imaging with PET/MRI. Further studies are needed to validate the management and cost-benefit aspects of this modality.

## Electronic supplementary material

Below is the link to the electronic supplementary material.

Supplementary material 1 (PPTX 636 kb)

## Abbreviations

LV: Left ventricle
LVEF: Left ventricular ejection fraction
PET: Positron Emission Tomography
CT: Computed Tomography
MRI: Magnetic Resonance Imaging
^18^F-FDG: Fluorine-18 fluorodeoxyglucose
LGE: Late Gadolinium Enhancement
