# Supplementary material for: Same day comparison of PET/CT and PET/MR in patients with cardiac sarcoidosis
Source: J Nucl Cardiol. 2019 Jan 2;27(6):2118–29. doi: 10.1007/s12350-018-01578-8 (PMC7749056; doi:10.1007/s12350-018-01578-8)
Supplement: Supplementary file 1 — Supplementary material 1 (PPTX 636 kb) [file 12350_2018_1578_MOESM1_ESM.pptx]

## Slide 1
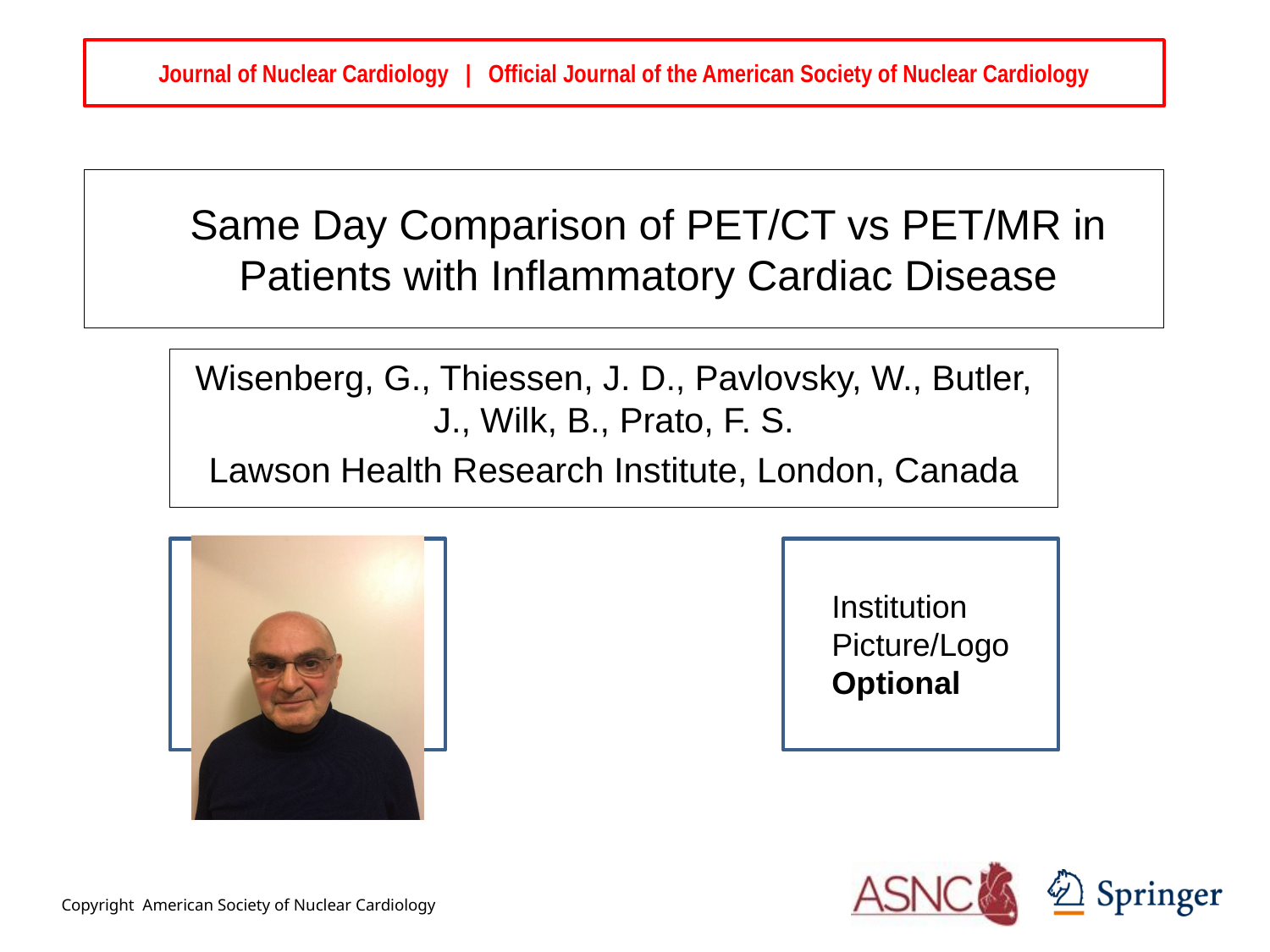

Journal of Nuclear Cardiology | Official Journal of the American Society of Nuclear Cardiology
# Same Day Comparison of PET/CT vs PET/MR in Patients with Inflammatory Cardiac Disease
Wisenberg, G., Thiessen, J. D., Pavlovsky, W., Butler, J., Wilk, B., Prato, F. S.
Lawson Health Research Institute, London, Canada
Head shot of author
required
Institution
Picture/Logo
Optional
Copyright American Society of Nuclear Cardiology

## Slide 2
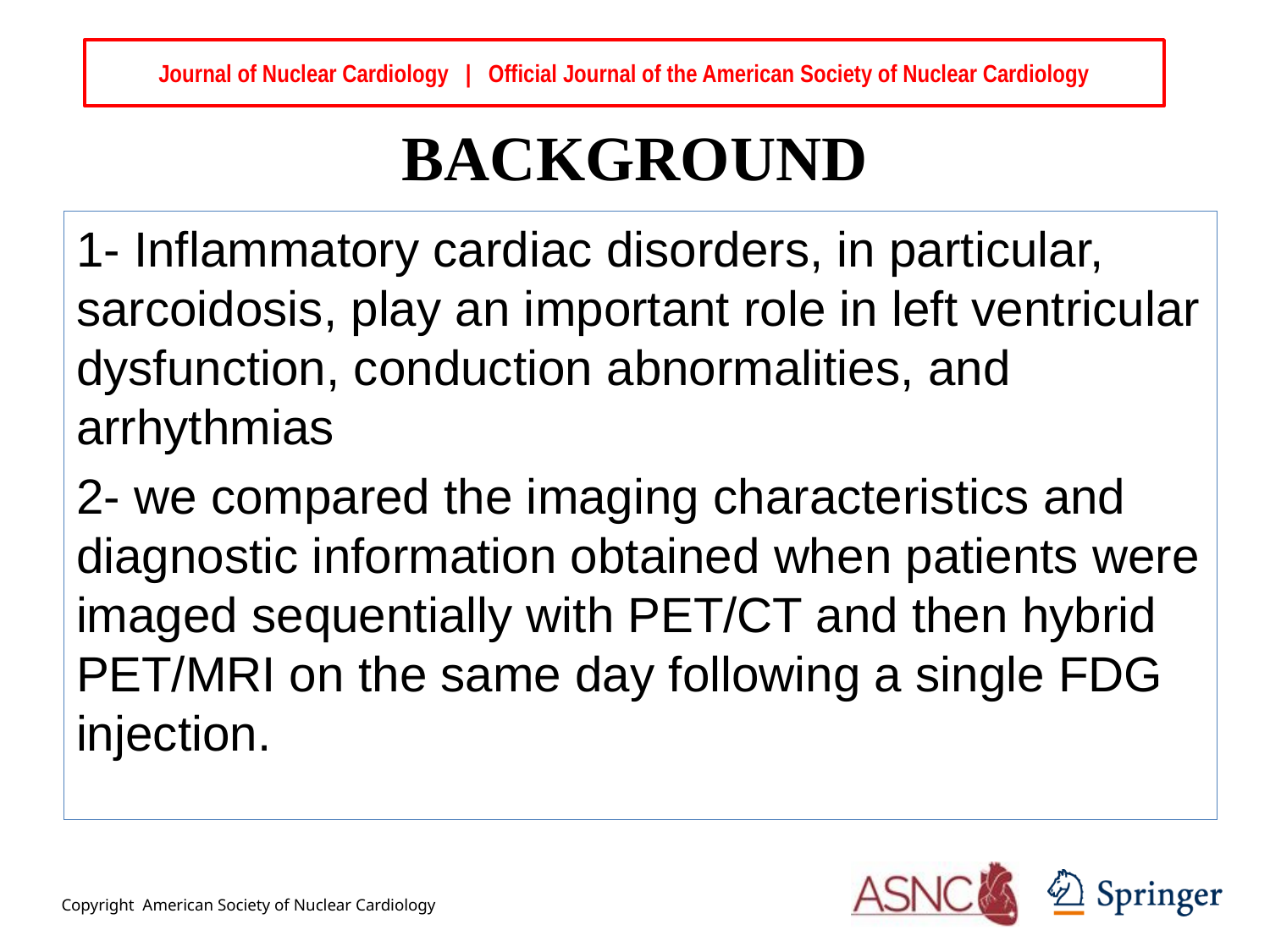

Journal of Nuclear Cardiology | Official Journal of the American Society of Nuclear Cardiology
# BACKGROUND
1- Inflammatory cardiac disorders, in particular, sarcoidosis, play an important role in left ventricular dysfunction, conduction abnormalities, and arrhythmias
2- we compared the imaging characteristics and diagnostic information obtained when patients were imaged sequentially with PET/CT and then hybrid PET/MRI on the same day following a single FDG injection.
Copyright American Society of Nuclear Cardiology

## Slide 3
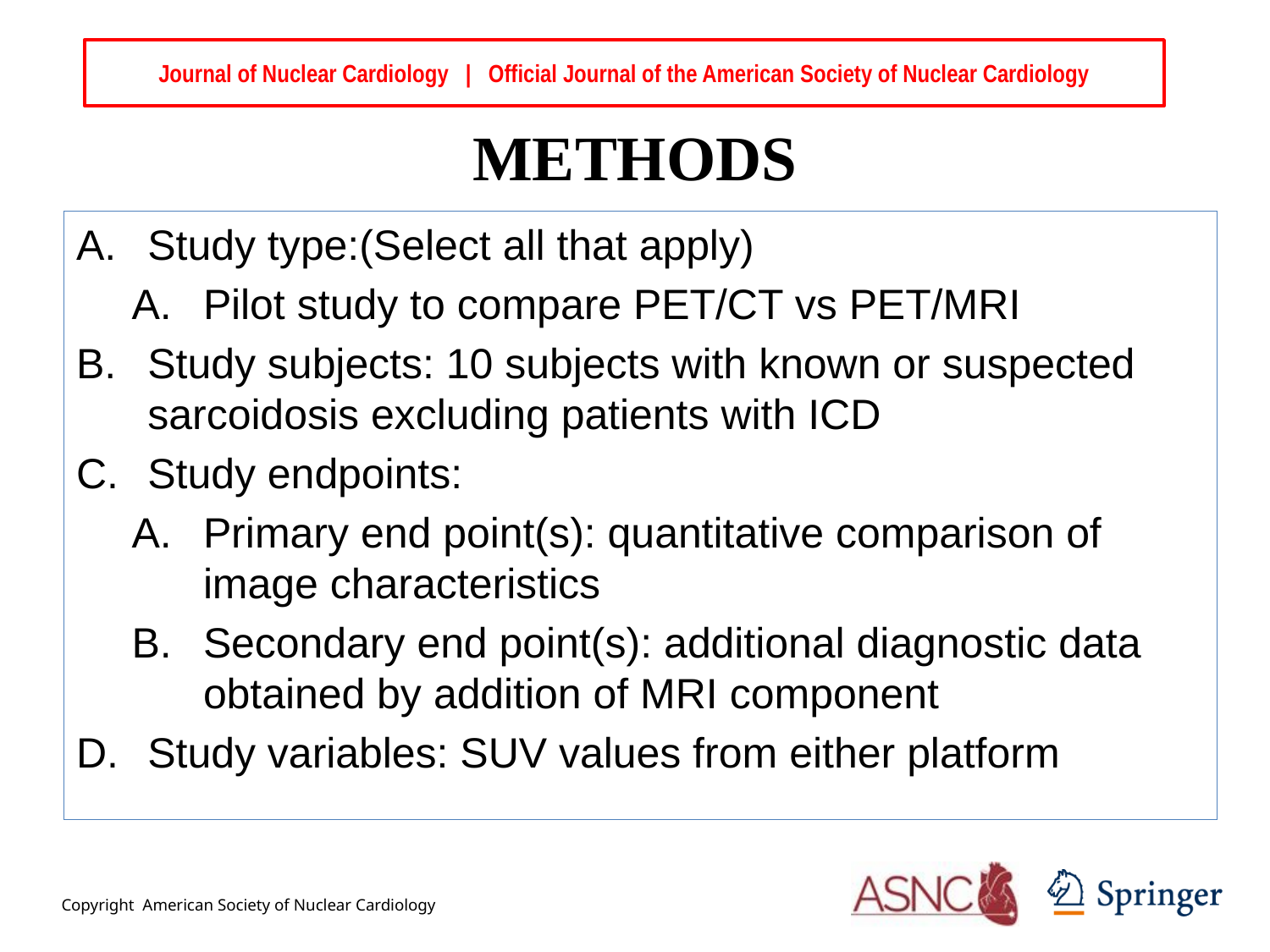

Journal of Nuclear Cardiology | Official Journal of the American Society of Nuclear Cardiology
# METHODS
Study type:(Select all that apply)
Pilot study to compare PET/CT vs PET/MRI
Study subjects: 10 subjects with known or suspected sarcoidosis excluding patients with ICD
Study endpoints:
Primary end point(s): quantitative comparison of image characteristics
Secondary end point(s): additional diagnostic data obtained by addition of MRI component
Study variables: SUV values from either platform
Copyright American Society of Nuclear Cardiology

## Slide 4
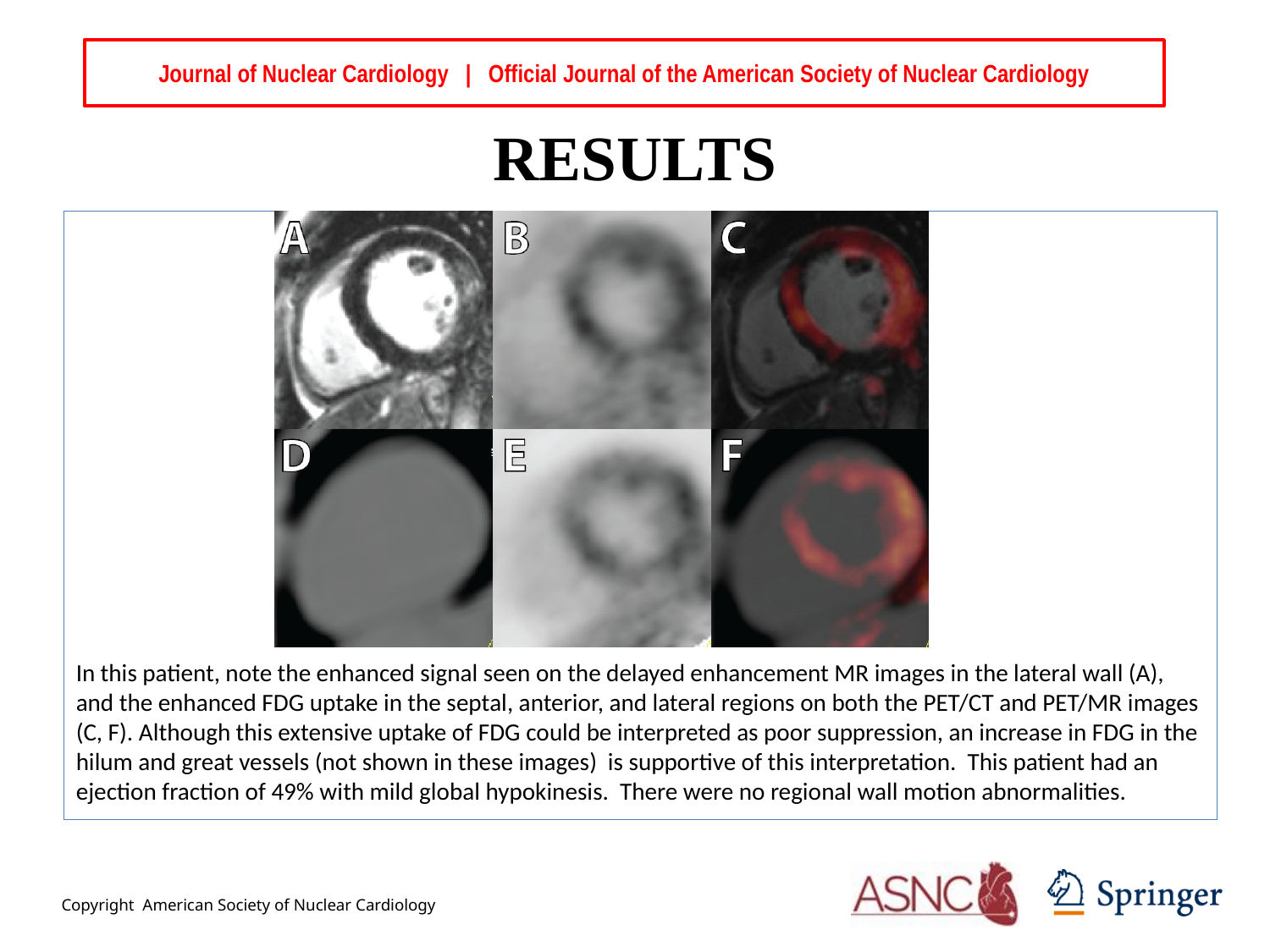

Journal of Nuclear Cardiology | Official Journal of the American Society of Nuclear Cardiology
# RESULTS
In this patient, note the enhanced signal seen on the delayed enhancement MR images in the lateral wall (A), and the enhanced FDG uptake in the septal, anterior, and lateral regions on both the PET/CT and PET/MR images (C, F). Although this extensive uptake of FDG could be interpreted as poor suppression, an increase in FDG in the hilum and great vessels (not shown in these images) is supportive of this interpretation. This patient had an ejection fraction of 49% with mild global hypokinesis. There were no regional wall motion abnormalities.
Copyright American Society of Nuclear Cardiology

## Slide 5
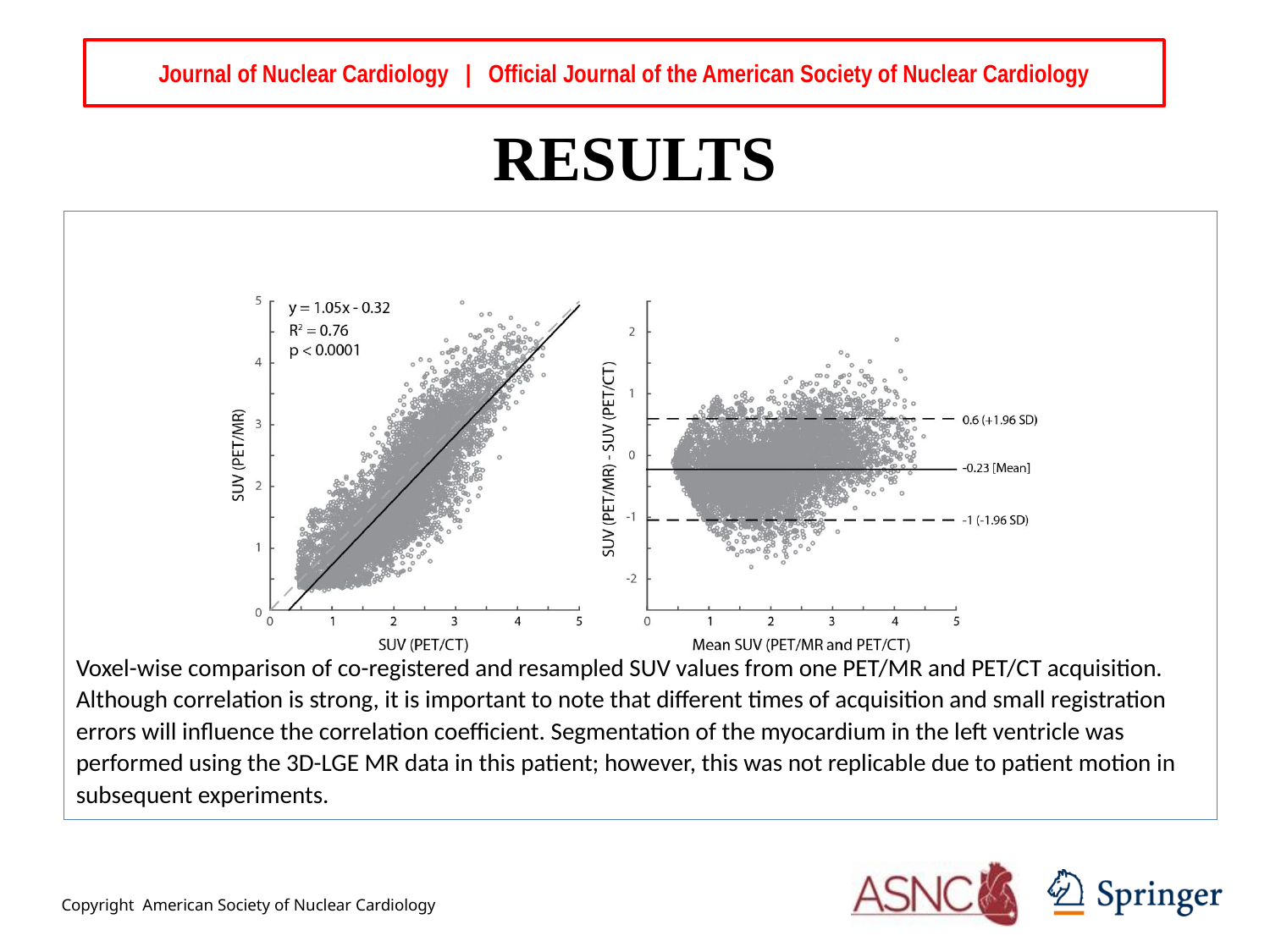

Journal of Nuclear Cardiology | Official Journal of the American Society of Nuclear Cardiology
# RESULTS
Insert a key table or a key figure
If figure, insert legend
Voxel-wise comparison of co-registered and resampled SUV values from one PET/MR and PET/CT acquisition. Although correlation is strong, it is important to note that different times of acquisition and small registration errors will influence the correlation coefficient. Segmentation of the myocardium in the left ventricle was performed using the 3D-LGE MR data in this patient; however, this was not replicable due to patient motion in subsequent experiments.
Copyright American Society of Nuclear Cardiology

## Slide 6
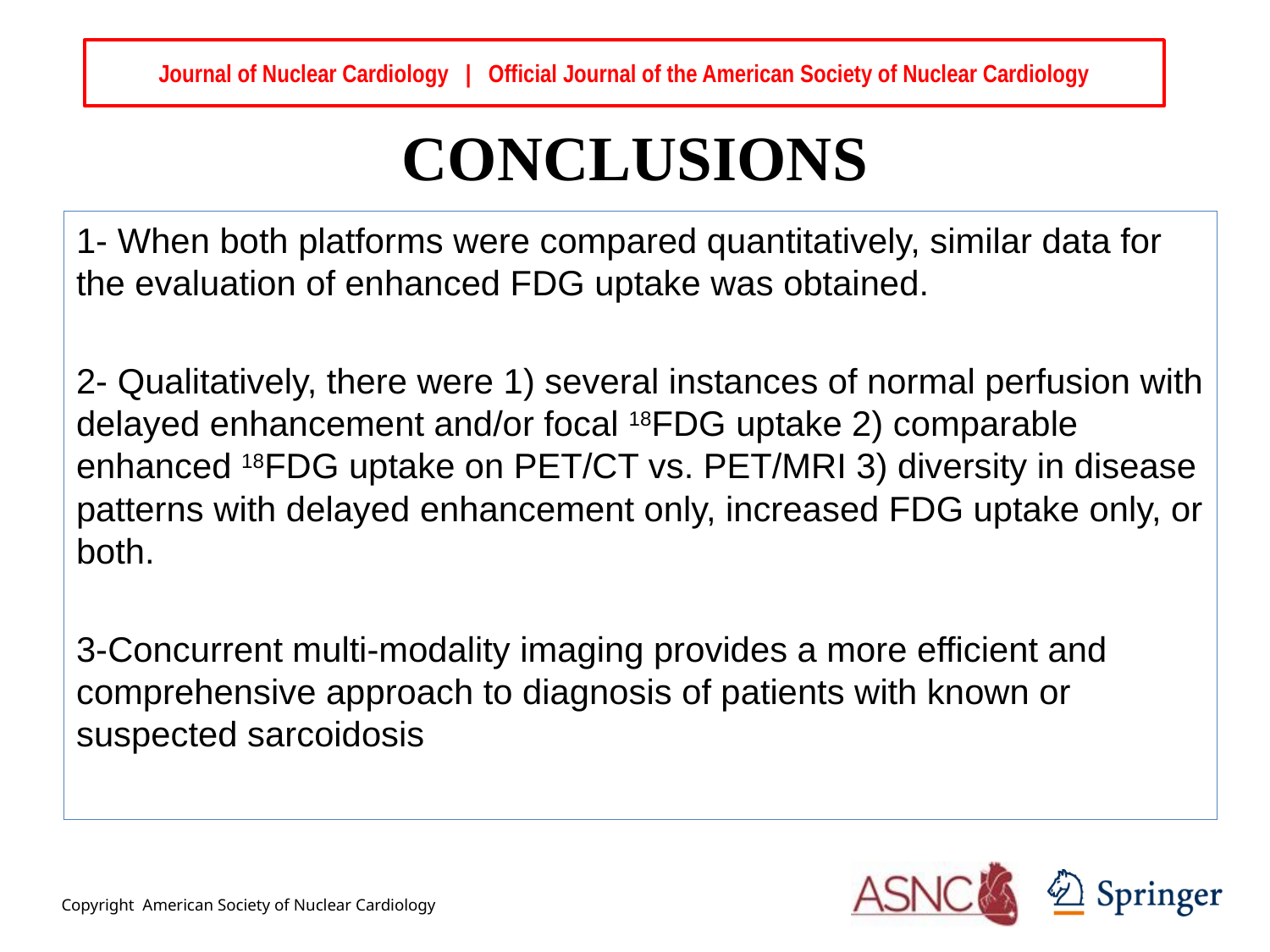

Journal of Nuclear Cardiology | Official Journal of the American Society of Nuclear Cardiology
# CONCLUSIONS
1- When both platforms were compared quantitatively, similar data for the evaluation of enhanced FDG uptake was obtained.
2- Qualitatively, there were 1) several instances of normal perfusion with delayed enhancement and/or focal 18FDG uptake 2) comparable enhanced 18FDG uptake on PET/CT vs. PET/MRI 3) diversity in disease patterns with delayed enhancement only, increased FDG uptake only, or both.
3-Concurrent multi-modality imaging provides a more efficient and comprehensive approach to diagnosis of patients with known or suspected sarcoidosis
Copyright American Society of Nuclear Cardiology
